# Green fluorescent protein as a reporter for the spatial and temporal expression of *actIII* in *Streptomyces coelicolor*

**DOI:** 10.1007/s00203-017-1358-1

**Published:** 2017-03-22

**Authors:** Fernando Santos-Beneit, Jeff Errington

**Affiliations:** Centre for Bacterial Cell Biology, Institute for Cell and Molecular Biosciences, Medical School, Newcastle University, Newcastle upon Tyne, UK

**Keywords:** Polyketides, Actinorhodin, *Streptomyces coelicolor*, ActIII, ActA, SCO5086, SCO5083

## Abstract

Polyketides constitute a large group of structurally diverse natural products with useful biological activities. Insights into their biosynthetic mechanisms are crucial for developing new structures. One of the most studied model polyketide is the blue-pigmented antibiotic actinorhodin, produced by *Streptomyces coelicolor*. This aromatic polyketide is synthesized by minimal type II polyketide synthases and tailoring enzymes. The ActIII actinorhodin ketoreductase is a key tailoring enzyme in actinorhodin biosynthesis. Previous papers have reported contradictory findings for localization of the protein in the cytoplasmic fraction or associated with the cell wall. We have now used green fluorescent protein as a reporter to analyse the spatial and temporal expression of *actIII* (SCO5086) in *S. coelicolor* under actinorhodin producing and non-producing conditions. We provide evidence in support of ActIII being a cytosolic protein, with limited if any association with the membrane or cell wall.

## Introduction

Polyketides are an important class of natural products with many different biological functions that can be produced by bacteria, fungi and plants (Hopwood 1997). The enzymes that synthesize these complex molecules are known as polyketide synthases. These proteins are analogous to the closely related fatty acid synthases which use the decarboxylation of α-carboxylated precursors to drive the synthesis of carbon chains (Keatinge-Clay et al. 2004). Actinorhodin provides a classic example of how these secondary metabolites are synthesized and regulated. This polyketide antibiotic is produced by the soil bacterium *Streptomyces coelicolor* and, as a blue isochromanequinone compound, provides a convenient model for studies of regulation (Rawlings 1999). The actinorhodin biosynthetic gene cluster (Act) has been extensively studied and the function of most genes characterized (Malpartida and Hopwood 1986; Fernandez-Moreno et al. 1994). The carbon backbone of actinorhodin is assembled through the cooperation of a ketosynthase/chain length factor (KS-CLF; *actI*-ORF1 and *actI*-ORF2), an acyl carrier protein (ACP; *actI*-ORF3), and a malonyl-CoA:ACP transacylase (MAT) which is shared between polyketide and fatty acid synthesis (Carreras and Khosla 1998; Dreier et al. 1999). The resulting backbone is tailored into actinorhodin by the action of various enzymes, including ketoreductases, cyclases, and oxygenases, which are important for generating structural diversity (Rix et al. 2002). The cellular locations of polyketide tailoring enzymes have not been investigated in detail, but they are assumed to reside in the cytoplasm or to be bound to the inner part of the membrane, because polyketide biosynthesis occurs intracellularly. Therefore, it is not clear if the detection of some polyketide tailoring enzymes such as the ActVI-ORF3 dehydrase (Hesketh et al. 2002) and the ActIII ketoreductase (Xu et al. 2008) in the cell-wall fraction of *S. coelicolor* should be considered as an anomaly or not. In particular, ActIII (SCO5086) has received a special attention due to its uncertain cellular localization. This protein, which has one predicted transmembrane segment, was found in the cytoplasmic fraction in a proteomic analysis by Hesketh and Chater (2003). However, Xu and co-workers (2008) were not able to detect the actinorhodin ketoredutase ActIII in the cytoplasmic protein fraction using Western blotting analyses. In contrast, after careful analysis of proteins in subproteomes, these authors found ActIII to be associated with the cell wall. To clarify the localization of this protein *in vivo*, we have examined the spatial and temporal expression pattern of *actIII* using the green fluorescence protein and epifluorescence microscopy. We also report a study of *actA* (SCO5083), which codes for one of the three putative export pumps identified within the *act* cluster (Tahlan et al. 2007), as a control for a peripheral membrane protein.

## Materials and methods

### Bacterial strains and plasmids

The bacterial strains, plasmids, and primers used in this work are listed in Table 1. *S. coelicolor* strains were manipulated according to the standard procedures (Kieser et al. 2000). *Escherichia coli* DH5α was the general cloning host. Cloning procedures were performed as described by Sambrook et al. (1989). The SCO5083 (*actA*) and SCO5086 (*actIII*) inserts were amplified by PCR using total DNA as template, as follows. The primers FSB66 and FSB67 amplified an 1894 bp fragment encompassing the *actA* ORF (not including the stop codon) and the promoter region of the gene from +1 to +125 (positions from the ATG translation start triplet). Primers FSB68 and FSB69 were used to amplify an 1762 bp fragment encompassing the *actIII* ORF (not including the stop codon) and the promoter region of the gene from positions +1 to +944 (positions from the translation start triplet). In both cases, the *Bgl*II (forward primer) and *Nde*I (reverse primer) cloning sites were introduced via the primer sequences. *Bgl*II–*Nde*I fragments were cloned into pIJ8660 (Sun et al. 1999) to obtain pSCO5083-EGFP and pSCO5086-EGFP, respectively. The inserts of both plasmids were checked by sequencing.

**Table 1:** Bacterial strains and plasmids used in this work

Strains	Characteristics	Reference or source
*S. coelicolor* M145	Wild type	Kieser et al. (2000)
*E. coli* DH5*α*	F′ Φ80 *dLacZ* ΔM15	Hanahan (1983)
*E. coli* ET12567	(pUZ8002) *dam dcm* mutant, Neo^r^-Cm^r^	MacNeil et al. (1992)
Plasmids		
pIJ8660	Integrative promoter-probe vector, EGFP gene, Am^r^	Sun et al. (1999)
pSCO5083-EGFP	*Bgl*II–*Nde*I SCO5083 fragment cloned into pIJ8660, Am^r^	This work
pSCO5086-EGFP	*Bgl*II–*Nde*I SCO5086 fragment cloned into pIJ8660, Am^r^	This work
Primers		
FSB66	5′CTTCAGATCTCTCGCTTCGCGACACGTGCTCCTCATCGTATG	This work
FSB67	5′GTCACCGCATATGGCCGCTCCCGGAGAAGCCCTCTTCCTCACGCGGCTTGGGCGGCAG	This work
FSB68	5′CAGGAGATCTGACGAACATCGCGGCTCCTTCGGCCAGCACGAAG	This work
FSB69	5′GTCACCGCATATGGCCGCTCCCGGAGTAGTTCCCCAGCCCGCCGCAGACGTTCAGCGC	This work

### Growth conditions

*Streptomyces coelicolor* solid cultures were performed in TSA (tryptone soya agar) using black polystyrene sterile 96-well plates. Each well (containing 100 µL of medium) was added 4 µL of a stock dilution containing 10^6^ spores of *S. coelicolor*. Plates were then incubated at 30 °C during 60 h. *S. coelicolor* liquid cultures were performed in defined MG-18.5 medium (Santos-Beneit et al. 2008). 100 ml of MG-18.5 medium in 500 ml baffled flasks were inoculated with 10^6^ spores per ml and incubated at 30 °C in an orbital shaker for reproducible and dispersed growth.

### Growth and antibiotic production determinations

Samples for growth and actinorhodin production determinations from the liquid cultures were taken after 26, 35, 46, 68, and 90 h of incubation. Growth was determined by dry weight (culture samples of 2 ml were washed twice with MilliQ water and dried for 4 days at 80 °C before the weight measurements). Growth in solid media was determined by optical density (420 nm) using a BMG Fluostar Optima fluorometer. Actinorhodin quantification was performed, as described by Kieser et al. (2000).

### Fluorescence intensities determination

The measurements were carried out at 30 °C using a BMG Fluostar Optima fluorometer as follows: Fluorescence Top Reading mode; multiple reads per well; excitation wavelength (485 nm); Emission wavelength (535 nm).

### Fluorescence microscopy

To visualise growing mycelia of *S. coelicolor* strains, cells were immobilized on microscope slides covered with a thin film of 1.2% agarose in water. Standard fluorescence microscopy was carried out using a Nikon Eclipse T*i* microscope with a Nikon Plan Fluor × 100/1.30 Oil objective. The images were acquired using MetaMorph (Molecular Devices) and further analysed using ImageJ (National Institutes of Health).

## Results and discussion

### Constructs for *actA* and *actIII*-EGFP fusion proteins

**Fig. 1:**
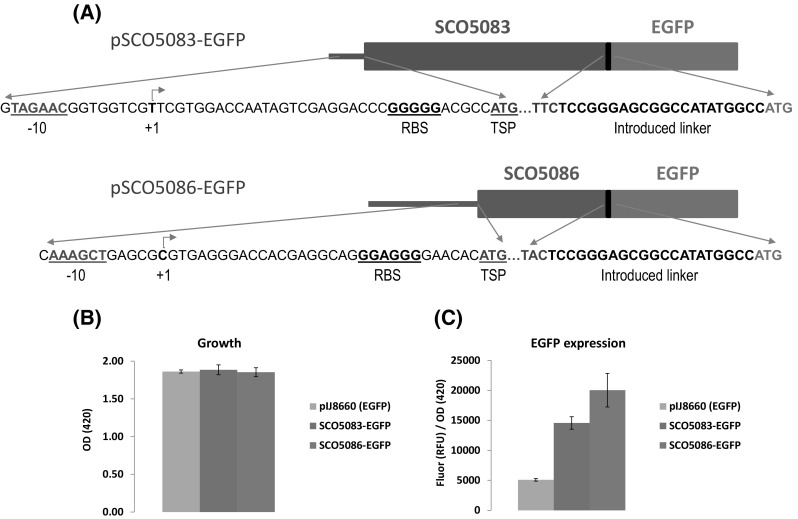
**a** Scheme of the SCO5083-EGFP and SCO5086-EGFP fusion protein constructions. The *black line* between the labelled coding regions corresponds to the introduced amino acid linker composed of Ser, Gly, Ser, Gly, His, Met, and Ala. RBS: ribosomal binding site. TSP: translation start point **b, c** Fluorescence reporter assay to quantify the expression of the SCO5083-EGFP and SCO5086-EGFP fusion proteins. Cells of the indicated strains were grown in TSA using black polystyrene sterile 96-well plates. Each well (containing 100 µL of medium) was inoculated with 4 µL of a stock dilution containing 10^6^ spores of *S. coelicolor*. Plates were then incubated at 30 °C for 60 h before being assayed for growth (**b**) or EGFP expression (**c**). *Vertical error bars* correspond to the standard error of the mean of 24 replicate cultures

To obtain a construct expressing *actA*-EGFP and *actIII*-EGFP, we used the integrative plasmid pIJ8660 (Sun et al. 1999), which contains a promoterless enhanced version of the GFP coding gene (EGFP). The SCO5083 (*actA*) and SCO5086 (*actIII*) genes, including their own promoters (Parro et al. 1991; Caballero et al. 1991), were amplified by PCR from *S. coelicolor* M145 total DNA. *Bgl*II and *Nde*I digested DNA fragments were then introduced in pIJ8660 to obtain pSCO5083-EGFP and pSCO5086-EGFP final vectors. For both SCO5083 and SCO5086 amplifications, the *Bgl*II restriction sequence was added to the 5′ region of the direct primer. In both cases, the reverse primers contained the last 33 bp of the ORF (not including the end codon) and a 5′ tail with the *Nde*I site. For both constructions, the tail contained the same sequence coding for a specific 6 aa-peptide. In this manner, the cloning of the respective fragments to the *Bgl*II/*Nde*I site of the vector left a Ser, Gly, Ser, Gly, His, Met, and Ala amino acid linker between the cloned gene and EFGP (note that there is an additional triplet for Ala in the pIJ8660 vector after the ATG of the *Nde*I site and before the start codon triplet of the EGFP; see Fig. 1a). A transcriptional terminator upstream of the cloning site avoids polar effects on the cloned genes, so that expression of the fusion proteins in pSCO5083-EGFP and pSCO5086-EGFP solely depends on the natural promoters of SCO5083 and SCO5086, respectively (see "Material and methods").

### SCO5083-EGFP and SCO5086-EGFP fusions are expressed in *S. coelicolor* TSA cultures

To check if the fusion proteins were indeed expressed, we made fluorescent reporter analyses in black polystyrene microtiter plates using a BMG Fluostar Optima fluorometer (see “Material and methods”). To carry out the assay, *S. coelicolor* spores (10^6^ in all cases) containing either pIJ8660, pSCO5083-EGFP or pSCO5086-EGFP vectors were grown for 60 h in TSA medium (supplemented with 50 µg/ml apramycin to maintain selective pressure on the plasmids), until actinorhodin production was evident. The growth yields at this timepoint were estimated by measuring OD_420_ in each well; mean values of 24 replicates for each of the strains used are shown in Fig. 1b. Protein expression was given in fluorescence specific values which were calculated as a ratio of relative fluorescence units and OD (Fig. 1c). Fluorescence values were 3–4 times higher in the strains carrying the fusion proteins than in the strain carrying the promoterless EGFP protein, indicating that both SCO5083-EGFP and SCO5086-EGFP fusions were expressed.

### Localization of SCO5083 (ActA) and SCO5086 (ActIII) proteins

As mentioned in the “Introduction ”, previous descriptions of the cellular localization of ActIII were conflicting. However, the membrane localization of ActA was expected to be unambiguously membrane associated as it is predicted to possess 13 transmembrane segments (Hesketh and Chater 2003). This protein was, therefore, expected to provide a membrane localization control for our analyses. The SCO5083-EGFP fluorescence signal should also demonstrate whether the fusion protein is stable and if the fusion protein linker is flexible enough to permit folding and activation of the fluorescent moiety.

**Fig. 2:**
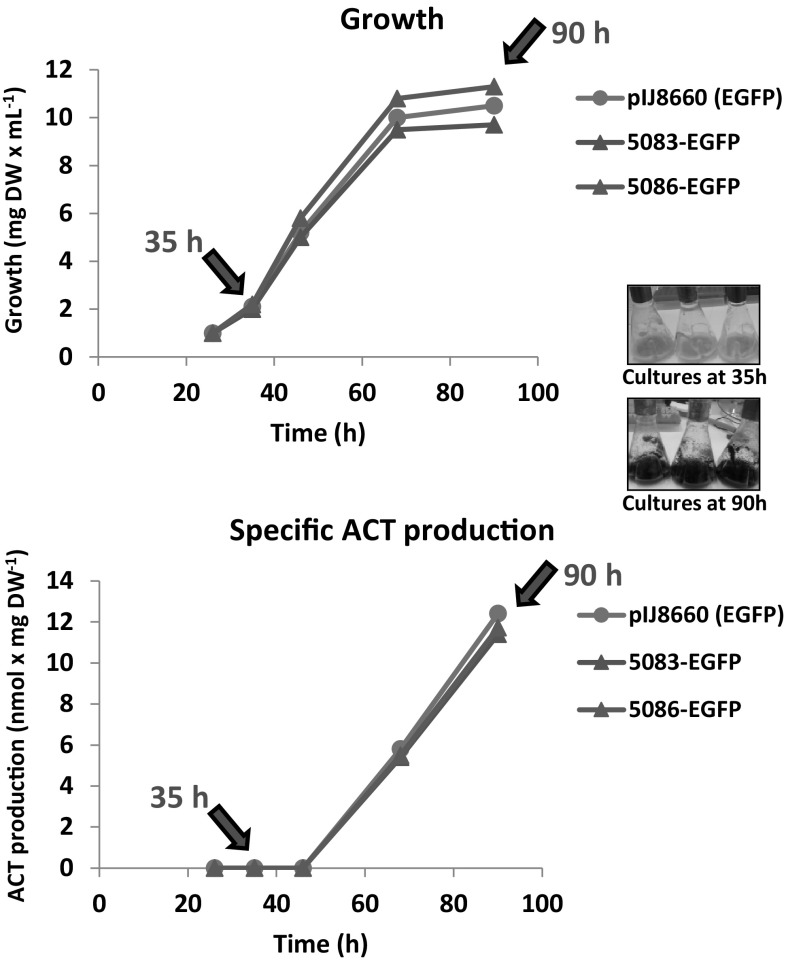
Growth (*upper panel*) and actinorhodin (ACT) production (*lower panel*) of *S. coelicolor* M145 strains carrying pIJ8660, pSCO5083-EGFP or pSCO5086-EGFP vectors. *Arrows* indicate the times at which samples for phase contrast and epifluorescence microscopy were taken

**Fig. 3:**
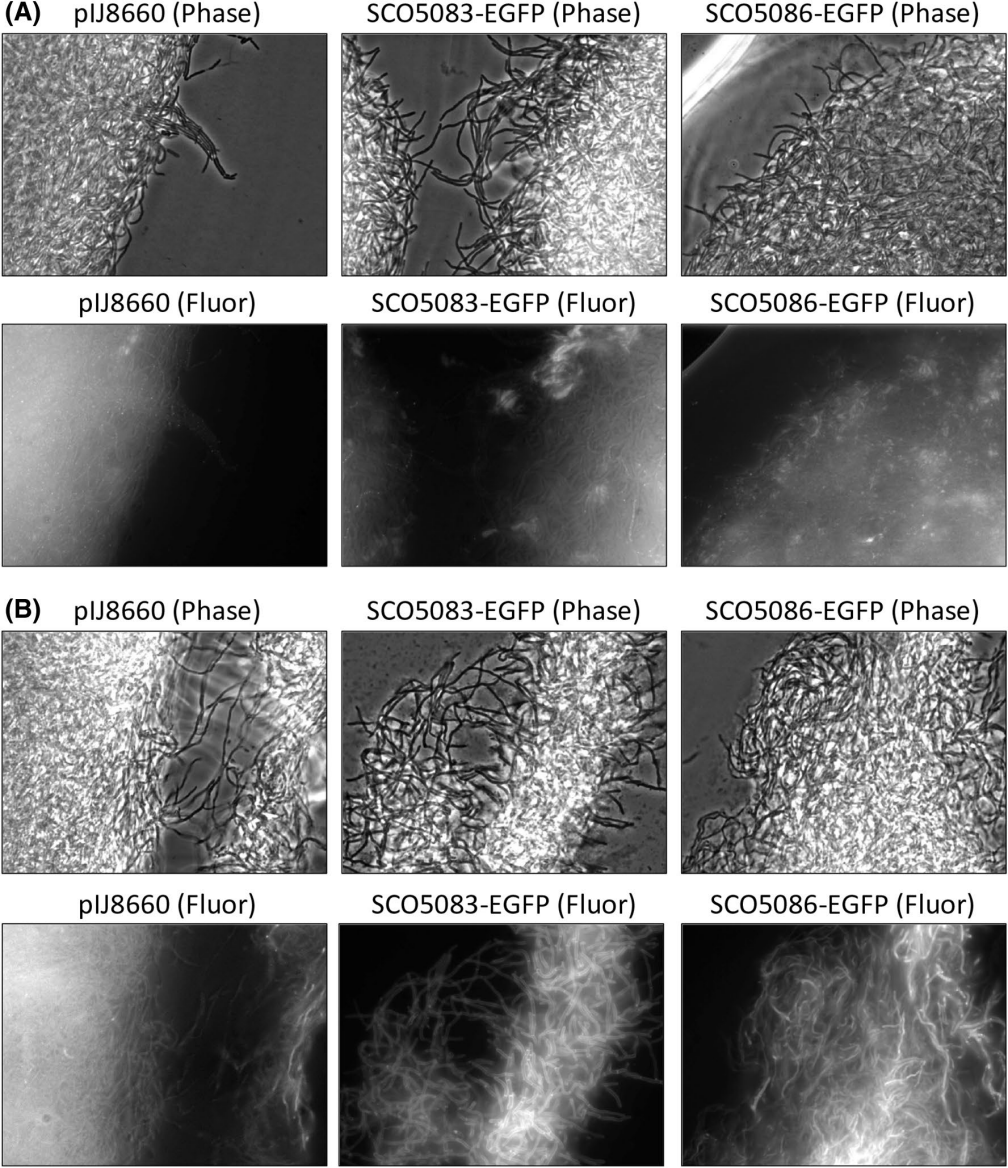
Localization of SCO5083-EGFP and SCO5086-EGFP expressed from their native locus in *S. coelicolor* M145. Cultures were incubated in MG-18.5 medium at 30 °C for 35 h (**a**) or 90 h (**b**). In each part, the *upper panels* correspond to phase contrast images and the *lower panels* to fluorescence images. Note that the exposure time for the fluorescence images in samples at 35 h was three times longer than in samples at 90 h (3000 and 1000 ms, respectively)

*Streptomyces coelicolor* cells containing pIJ8660, pSCO5083-EGFP, and pSCO5086-EGFP were grown in MG-18.5 medium (Santos-Beneit et al. 2008) in which undecylprodigiosin production (the other pigmented antibiotic produced by *S. coelicolor*) is almost suppressed, but not actinorhodin (Santos-Beneit et al. 2009). Growth and actinorhodin production were monitored during the time course of the experiment to identify samples corresponding to the actinorhodin production phase (see Fig. 2). Samples were selected at 35 h (production off) and 90 h (production on). No significant differences in fluorescence were observed between the 3 strains in the 35 h samples (see Fig. 3a). However, fluorescence was much higher in the cells containing either pSCO5083-EGFP or pSCO5086-EGFP when the samples at 90 h were analysed (even using three times lower exposure times than in the 35 h samples; see Fig. 3b). The negative control (*S. coelicolor* harbouring the pIJ8660 vector), which grew and produced actinorhodin similarly to the two other strains (see Fig. 2), failed to show any increase in the fluorescence signal. This result was in agreement with the temporal expression patterns expected for ActIII and ActA; i.e., the proteins should only be expressed during the actinorhodin production phase.

**Fig. 4:**
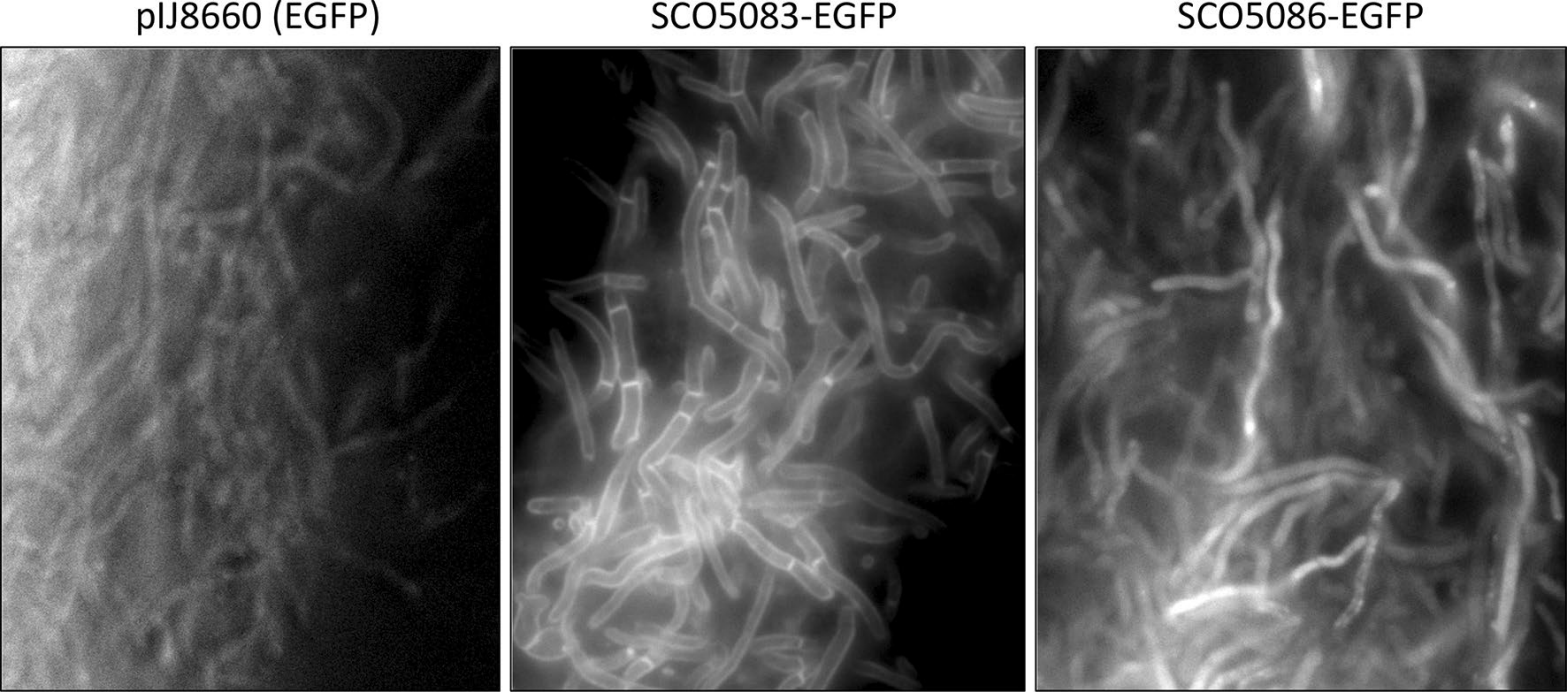
Detail of EGFP expression in *S. coelicolor* M145 cells, containing pIJ8660 (control) pSCO5083-EGFP and pSCO5086-EGFP vectors, cultures in MG-18.5 medium for 90 h. All of the fluorescence images were obtained with an exposure time of 1000 ms

In relation to the spatial expression patterns, the SCO5083-EGFP fusion protein localized specifically to the membrane of the *S. coelicolor* cells, including not only the periphery of the cells but also the vegetative cross-walls (see Fig. 4). However, the localization of the pSCO5086-EGFP fusion protein was completely different to that of pSCO5083-EGFP; the fluorescence was observed within the cell body rather than only in the periphery. This fluorescence pattern for pSCO5086-EGFP strongly suggested that ActIII is a cytosolic protein, with limited if any association with the membrane or cell wall. We cannot formally exclude the possibility that the native (i.e., non-EGFP) SCO5086 protein could have a different localization pattern but given that the pool of the wild type and fusion proteins supports the same actinorhodin production yields than the wild-type protein, it seems that the cytosolically localized protein is functional.
